# Skeleton optimization of neuronal morphology based on three-dimensional shape restrictions

**DOI:** 10.1186/s12859-020-03714-z

**Published:** 2020-09-04

**Authors:** Siqi Jiang, Zhengyu Pan, Zhao Feng, Yue Guan, Miao Ren, Zhangheng Ding, Shangbin Chen, Hui Gong, Qingming Luo, Anan Li

**Affiliations:** 1 Britton Chance Center for Biomedical Photonics, Wuhan National Laboratory for Optoelectronics, MoE Key Laboratory for Biomedical Photonics, School of Engineering Sciences, Huazhong University of Science and Technology, Wuhan, China; 2 School of Biomedical Engineering, Hainan University, Haikou, China; 3 HUST-Suzhou Institute for Brainsmatics, JITRI Institute for Brainsmatics, Suzhou, China; 4 CAS Center for Excellence in Brain Science and Intelligence Technology, Chinese Academy of Science, Shanghai, China

**Keywords:** Neuronal morphology, Neuron tracing, Neuronal skeleton optimization, Shape restriction

## Abstract

**Background:**

Neurons are the basic structural unit of the brain, and their morphology is a key determinant of their classification. The morphology of a neuronal circuit is a fundamental component in neuron modeling. Recently, single-neuron morphologies of the whole brain have been used in many studies. The correctness and completeness of semimanually traced neuronal morphology are credible. However, there are some inaccuracies in semimanual tracing results. The distance between consecutive nodes marked by humans is very long, spanning multiple voxels. On the other hand, the nodes are marked around the centerline of the neuronal fiber, not on the centerline. Although these inaccuracies do not seriously affect the projection patterns that these studies focus on, they reduce the accuracy of the traced neuronal skeletons. These small inaccuracies will introduce deviations into subsequent studies that are based on neuronal morphology files.

**Results:**

We propose a neuronal digital skeleton optimization method to evaluate and make fine adjustments to a digital skeleton after neuron tracing. Provided that the neuronal fiber shape is smooth and continuous, we describe its physical properties according to two shape restrictions. One restriction is designed based on the grayscale image, and the other is designed based on geometry. These two restrictions are designed to finely adjust the digital skeleton points to the neuronal fiber centerline. With this method, we design the three-dimensional shape restriction workflow of neuronal skeleton adjustment computation. The performance of the proposed method has been quantitatively evaluated using synthetic and real neuronal image data. The results show that our method can reduce the difference between the traced neuronal skeleton and the centerline of the neuronal fiber. Furthermore, morphology metrics such as the neuronal fiber length and radius become more precise.

**Conclusions:**

This method can improve the accuracy of a neuronal digital skeleton based on traced results. The greater the accuracy of the digital skeletons that are acquired, the more precise the neuronal morphologies that are analyzed will be.

## Background

Neurons are the basic structural unit of the brain. Their morphology is a key determinant of neuron type classification [1–5]. The morphology of a neuronal circuit is a fundamental component for neuron modeling [6]. With improvements in sparse labeling and optical microscopy techniques, scientists are able to acquire three-dimensional whole-brain images at a submicron-resolution for mammals [7–11]. From these images, the techniques that accurately transform them into digital descriptions of neuron morphology are very important; this transformation process is called neuron tracing. Neuron tracing is an important technique in neuroscience that includes many automatic tracing methods, such as skeletonization [12, 13], minimum spanning trees [14], snake models [15, 16], principle curve models [17] and neural network methods [18]. These automatic tracing methods are always used in small image blocks and work well. However, when dealing with a whole-brain image dataset, researchers prefer semimanual tracing [2, 8, 19–21] (researchers manually mark the neuron fiber nodes according the images, and software automatically links these nodes to create a neuron morphology file).

Existing neuron tracing studies mostly focus on the correctness and completeness of neuronal morphology. For neuron tracing from whole-brain images, the results of semimanual tracing are credible, so they are chosen as the ground truth in many studies to evaluate automatic method performance [17, 22]. However, in a semimanually traced neuronal skeleton, we note some inaccuracies. The distance between consecutive nodes is very long, spanning multiple voxels. The nodes are marked around the centerline of the neuronal fiber, not on the centerline. Although these inaccuracies will not seriously affect the topology of the neuronal morphology file, they will reduce the accuracy of the traced neuron skeleton. Since the ability of people to distinguish local voxels with little difference in grayscale is not as accurate and objective as that of a computer, inaccuracies generally appear in semimanually traced neuronal skeletons and may also appear in some automatically traced neuronal skeletons. These small inaccuracies will introduce deviations into subsequent tasks based on neuronal morphology files, such as neuronal fiber radius estimation and neuronal fiber length calculation. At present, with the emergence of more long-projection neuronal skeletons, it is urgent for us to improve the accuracy of these neuronal skeletons.

Here, we propose a neuronal digital skeleton optimization method to adjust a traced neuronal skeleton to the centerline of the neuronal fiber in images. This method evaluates and finely adjusts the skeleton according to three-dimensional shape restrictions, which are designed on the basis of the smooth and continuous properties of neuronal fibers. This method reduces the distance between the traced neuronal skeleton and the centerline of the neuronal fiber in images, thereby increasing the accuracy of the traced neuronal skeleton. Furthermore, based on the optimized skeletons, we calculate the neuronal morphology parameter to evaluate its effect. The results show that the more accurate the skeleton is, the more the accuracy of morphology metrics, such as neuronal fiber lengths and radiuses, are improved.

## Methods

### Dataset and computing environments

We used two datasets for testing. The first is provided by Wang et al. [11], and it consists of genetic labeling chandelier cells imaged by the fMOST system with a resolution of 0.2 × 0.2 × 1 μm^3^. The second is provided by Zhang et al. [23] and Jiang et al. [24], and it consists of pyramidal neurons at the same resolution. Two mice were used, one for the chandelier cells and the other for the pyramidal neurons. The mice were anesthetized deeply using a 1% solution of sodium pentobarbital and sacrificed by injection of ketamine. The semiautomatic neuron tracing used in the quantitative evaluation was performed with Amira software (version 6.1.1; FEI, Mérignac Cedex, France); the code of this method was written in MATLAB (R2019a, MathWorks) and run on a graphics workstation (Xeon CPU E5–2687 v3).

### Workflow of the proposed method

**Fig. 1:**
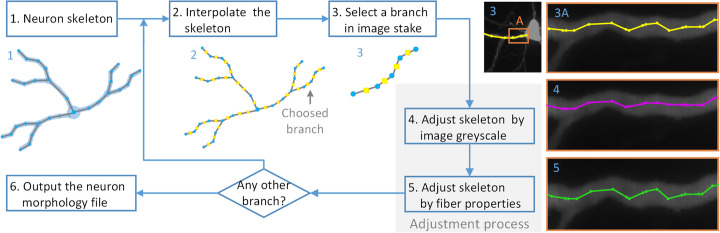
The computational workflow of skeleton optimization. The first stage is the preprocessing of the skeleton for optimization. The first stage is to interpolate the digital skeleton to increase the points and extract a branch from the digital skeleton; this includes steps 1 to 3. The second stage is digital skeleton optimization, which includes steps 4 and 5. The last stage is to output the neuron morphology file

Figure 1 depicts the computational workflow, including its three stages. The skeleton optimization method is the second stage, and the first and third stages are the pre- and postprocessing of the neuronal morphology, respectively.

The first phase is to extract a branch from the digital skeleton of the tree structure (Steps 1–3 in Fig. 1). Specifically, we linearly interpolate the digital skeleton and select the branch to be adjusted. The second stage involves fine-tuning the points derived from the digital skeleton to the centerline of the neuronal fibers from the images (Steps 4–5 in Fig. 1). Specifically, we initially adjust the digital skeleton points that are far from the centerline of the neuronal fibers based on the image grayscale. Subsequently, provided that the neuron fiber shape is smooth and continuous, we describe this physical property with two shape restrictions. One restriction is designed based on the image grayscale: the difference in the signal brightness between the local voxels around the neuronal fiber centerline must be small. The other is designed based on geometry: the angle formed from three continuous points on the neuronal fiber centerline must not be extreme. These two restrictions are designed to finely adjust the digital skeleton points to the neuronal fiber centerline. Subsequently, we join the branches into a tree structure according to the connected relationships, which comprises the third stage (Step 6 in Fig. 1). Finally, the digital skeleton yields a digital neuron morphological file.

With regard to the above three stages, the second stage is a looping structure. See the flowchart in Fig. 1 for details.

### Skeleton point adjustment based on grayscale

According to the grayscale distribution of the voxels around the neuronal fibers, a preliminary adjustment of the digital skeleton points that are far away from the neuronal fiber centerline shown in the image is performed. We take advantage of the idea of using the mean shift to find the location of this centerline point. Because of anisotropy in the image stacks, we linearly interpolate the three-dimensional images in the *Z* direction to obtain images with the same resolution in the *X*, *Y* and *Z* directions. For a skeleton point *p*_0_(*x*_0_, *y*_0_, *z*_0_), take an adjustment in the *Z* direction as an example. We keep the *X* and *Y* coordinates unchanged and calculate the convolution of the voxels in the local range centered on *p*_0_ and the Gaussian kernel function in the *Z* direction. The point that has the largest convolution value is regarded as the centerline point *p*_1_ of the neuronal fibers from the images.

1 $$ {p}_1=\arg \underset{p}{\max } Gau\otimes G\left({p}_0\right) $$

Similarly, the *X* and *Y* coordinates of the skeleton point *p*_1_ are adjusted to the centerline point to finally obtain the adjusted point *p*_2_.

### Skeleton point adjustment based on shape restrictions

To describe the tortuous neuronal fiber morphology with more accurate digital skeletons, it is necessary to fine-tune the skeleton points after the preliminary adjustment. The neuronal fibers are smooth and continuous, and this neuronal fiber property is shown in the images. Considering this property, for the point *p*_2_, we designed an angle restriction and brightness restriction to judge whether *p*_2_ needs further fine adjustment. Specifically, for *p*_2_, we determined its parent point *p*_*f*_, the parent point of *p*_*f*_, *p*_*ff*_, and the child point *p*_*c*_. The point *p*_2_ forms an angle ∠*p*_*f*_*p*_2_*p*_*c*_ with *p*_*f*_ and *p*_*c*_. Because the neuron fibers are smooth, the angle ∠*p*_*f*_*p*_2_*p*_*c*_ tends to be close to 180°. We set the critical value of the angle *α*_*std*_ as an obtuse angle (135° is chosen in this dataset). If ∠*p*_*f*_*p*_2_*p*_*c*_ < *α*_*std*_, *p*_2_ is considered to require further adjustment. For the brightness restriction, we compared the signal strength ${G}_{p_2}$ at point *p*_2_ with the signal strength threshold *G*_*std*_, thus designing *G*_*std*_ as follows:

2 $$ {G}_{std}=\max \left(\left({G}_{p_f}+{G}_{p_2}+{G}_{p_c}\right)/3,\kern0.5em {{G_B}_{ranch}}_{\_ mean}+{G}_{Branch\_ stdev}\right) $$

Here, ${G}_{p_f}$ is the signal strength of *p*_*f*_, taking the grayscale value of *p*_*f*_ in the same way as for ${G}_{p_2}$ and ${G}_{p_c}$. *G*_*Branch* _ *mean*_ and *G*_*Branch* _ *stdev*_ are the mean and standard deviation of the signal strength values of all points on the current branch. If ${G}_{p_2}>{G}_{std}$, the signal strength of *p*_2_ is in accordance with that of the current branch. However, if ${G}_{p_2}<{G}_{std}$, *p*_2_ is identified as a point that requires further adjustment. If its signal strength is small, this is contrary to the fact that the signal strength at the center of the neuronal fiber in images tends to be relatively strong.

According to these restrictions, we set out to find the centerline points of the neuronal fiber from the images. Prior to adjusting *p*_2_, because *p*_2_ itself may be a biased point, we have to judge whether we need to find a new starting point other than *p*_2_ for subsequent computation. Here, if ∠*p*_*f*_*p*_2_*p*_*c*_ > *α*_*std*_ and ${G}_{p_2}<{G}_{std}$, then there is only a problem with the brightness of point *p*_2_. In this situation, the starting point *p*_2 _ *new*_ will be equal to *p*_2_. If ∠*p*_*f*_*p*_2_*p*_*c*_ < *α*_*std*_, this indicates that there is a serious deviation in the angle pertaining to *p*_2_, and such a point is likely to have a problem with both brightness and angle. Therefore, we have to find a new starting point *p*_2 _ *new*_. The assumption here is that $\left\Vert \overrightarrow{p_2{p}_f}\right\Vert <\left\Vert \overrightarrow{p_2{p}_c}\right\Vert$. In the $\overrightarrow{p_2{p}_c}$ direction, with *p*_2_ as the starting point, we draw $\left\Vert \overrightarrow{p_2{p}_{rc}}\right\Vert =\left\Vert \overrightarrow{p_2{p}_f}\right\Vert$ and obtain a point *p*_*rc*_ on $\overrightarrow{p_2{p}_c}$. The intersection of the vertical bisector of $\overrightarrow{p_f{p}_{rc}}$ and $\overrightarrow{p_f{p}_{rc}}$ is defined as the point *p*_*mid*_. In the $\overrightarrow{p_f{p}_{ff}}$ direction, we draw $\left\Vert \overrightarrow{p_f{p}_s}\right\Vert =\left\Vert \overrightarrow{p_2{p}_f}\right\Vert$ and obtain a point *p*_*s*_ on the extension line of $\overrightarrow{p_f{p}_{ff}}$ (the topological graph is shown in Figure S1 A of the supplementary materials). Then, *p*_2 _ *new*_ is located as:

3 $$ {p}_{2\_ new}=\left({G}_{p_{mid}}\cdot {p}_{mid}+{G}_{p_2}\cdot {p}_2+{G}_{p_s}\cdot {p}_s\right)/\left({G}_{p_{mid}}+{G}_{p_2}+{G}_{p_s}\right) $$

Here, ${G}_{p_{mid}}$ represents the signal strength of *p*_*mid*_, taking the grayscale value of *p*_*mid*_ in the same way as for ${G}_{p_2}$ and ${G}_{p_s}$. With the starting point *p*_2 _ *new*_, we use the idea of the mean drift to search for the centerline points of the neuronal fiber from the images. The drift direction ${\overset{\rightharpoonup }{\upsilon}}_2$ in a spherical region *S*_*p*_ centered on *p*_2 _ *new*_ is calculated as follows:

4 $$ {\overset{\rightharpoonup }{\upsilon}}_2=\sum \limits_{p_i\in {S}_p}{a}_i\cdot {b}_i\cdot \overrightarrow{p_i{p}_{2\_ new}}/\left\Vert \overrightarrow{p_i{p}_{2\_ new}}\right\Vert $$

where *p*_*i*_ is the *ith* voxel in the spherical region *S*_*p*_. ${a}_{p_i}$ is the angle factor of *p*_*i*_, ${a}_{{\mathrm{p}}_i}=\sin {\theta_{p_i}}^2$, where ${\theta}_{p_i}$ is the angle between the vectors $\overrightarrow{p_f{p}_{rc}}$ and $\overrightarrow{p_{2\_ new}{p}_i}$, and ${b}_{p_i}$ is the signal strength factor of *p*_*i*_, ${b}_{p_i}=\exp \left(-{\left({G}_{p_i}-\mu \right)}^2/2{\sigma}^2\right)-\exp \left(-{\left({G}_{p_{2\_ new}}-\mu \right)}^2/2{\sigma}^2\right)$, where , and $\sigma =\left|\mu -{G}_{p_{2\_ new}}\right|$.

In the ${\overset{\rightharpoonup }{\upsilon}}_2$ direction, we search for the point

5 $$ {p}_3=\arg \max \left({\omega_1}^{\left\Vert \overrightarrow{p_i{p}_{2\_ new}}\right\Vert }{G}_{std}\sqrt{G_{p_i}/{G}_{std}}/{G}_{p_{2\_ new}}-{\omega}_2\cos \angle {p}_f{p}_{2\_ new}{p}_c\cos \angle {p}_f{p}_i{p}_c\right) $$

${G}_{p_i}$ is the signal strength at voxel *p*_*i*_, taking the grayscale value of *p*_*i*_. ${G}_{p_{2\_ new}}$ is the signal strength of *p*_2 _ *new*_. This means that *ω*_1_ and *ω*_2_ are the parameters that can be adjusted according to the image quality. When the parameter *ω*_1_ is set to a larger value, this means that we regard the grayscale value as a large influence on the points. However, when *ω*_2_ is set to a larger value, this means that we regard the angle deviation of the points as having a greater influence.

### Quantitative evaluation on the synthetic dataset

**Fig. 2:**
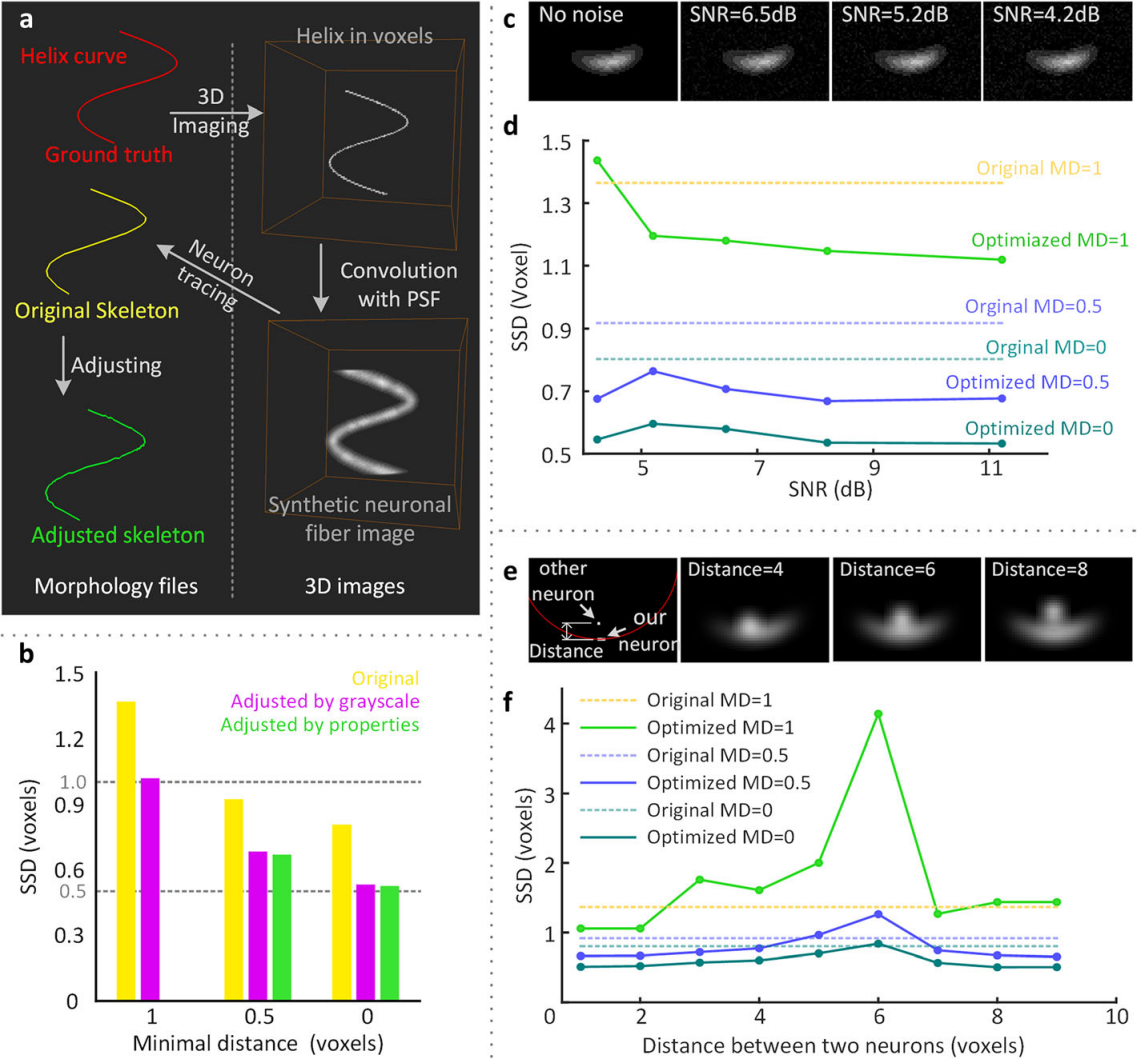
Quantitative performance of the skeleton optimization method on synthetic datasets. **a** The creation of the synthetic datasets and the ground truth. **b** Comparison of the adjusted skeletons and original skeleton. The cross-sections of the synthetic neuron fibers are shown under different SNRs (**c**) and at different distances from the adjacent neuron fiber (**e**). Skeleton optimization performance on datasets with different signal-to-noise ratios (**d**) and nearby neurons (**f**)

Since the goals of most automatic neuron tracing methods are different from ours, the semimanually traced neuron skeleton, which is regarded as the ground truth in most studies, is one of our targets that needs to be optimized. Therefore, we have to find a ground truth, rather than the semimanually traced results, to evaluate the performance of our methods. Here, we design a three-dimensional synthetic neuron fiber image to evaluate our methods (Fig. 2). First, we choose a helix curve (t∈(π,3π], x = 30 × sin(t) + 60, y = 30 × cos(t) + 60, z = 10 × t) as the centerline of the neuron fiber, which is the ground truth of the traced neuron skeleton. Then, in three-dimensional images, we use continuous voxels to represent this helix curve. Next, we choose the Gaussian function as the point spread function (PSF) of the imaging system and convolve it with the helix curve images. The result simulates the neuron fiber images. Based on these images, we semimanually trace the neuron fibers. Following a previous procedure [8, 11, 19], the semimanually traced neuron skeleton is completed interactively in Amira visualization and data analysis software. The skeleton is checked back-to-back by 3 people. The traced skeleton is regarded as the optimization target. Finally, we use our method to optimize the skeleton.

The noise of nearby neuron fibers and other fibers can affect the quality of the automatically traced neuron skeleton. To evaluate the performance of our method in such cases, we add Gaussian noise with different signal-to-noise ratio levels (Fig. 2c and d). For the case of nearby neurons, we set a straight line as representing another neuron whose distance from the helix curve changes (Fig. 2e and f).

In the research on automatic neuron tracing methods, there are various parameters used to evaluate the traced results from different perspectives [22, 25, 26]. Among these parameters, the significant spatial distance (SSD) is a parameter that can intuitively evaluate the distance between two neuron skeletons, and it is suitable for us to use to quantitatively evaluate the difference between the traced neuron skeleton and the ground truth. The SSD is defined as the average distance of the reciprocal minimal spatial distances between two neuron skeleton nodes. A larger SSD means that these two neuron skeletons are more different from each other. In Peng’s work [22], if the distance between two nodes is less than two pixels, it is difficult to determine the difference visually. As a result, when calculating the SSD, the minimum reciprocal distance is set to 2 pixels. However, in our experiment, the maximum reciprocal distance between the unoptimized skeleton and the ground truth is less than 2 voxels. Therefore, we set the minimum reciprocal distance to 1 voxel. In addition, we also provide the SSDs that the minimum reciprocal distances are set to 0 and 0.5 voxels.

### Process for a whole-brain three-dimensional image dataset

We used image blocks from TDat [27] and converted the three-dimensional image dataset, which is 32,266 × 54,600 × 10,730 voxels (more than 30 TB) with a depth of 16 bit/voxel, to the TDat data format. TDat divides a three-dimensional image dataset into many small three-dimensional image blocks. We then split the reconstructed digital skeleton into many fragments according to the range of each TDat image block, and the skeleton fragments in each block were calculated in turn. Finally, the fragments were connected to become a complete digital skeleton. This process was slightly different from the workflow for the local neuronal fibers. In summary, for the locally distributed neuronal fibers, we split the digital skeletons according to the branch structure. However, for the long-projection neuronal fibers, we split the digital skeletons according to the range of the blocks.

### Neuronal fiber radius estimation

In a real dataset, it is difficult to obtain the centerline of the neuron fiber images. Thus, we do not have a way to directly evaluate the difference between the traced neuron skeletons and the ground truth. However, according to the traced neuron skeleton, we can calculate the neuron morphology parameters to evaluate the differences in the sizes. Here, we choose the neuron fiber length and neuron fiber radius to evaluate the performance of our method.

The estimation process for the neuron fiber radius is as follows (see Fig. 5): Based on the topology of the digital skeleton, we calculate a cross-sectional plane of a skeleton point. Thereafter, with a series of image processing operations, this plane is used to segment the area of the neuronal fiber tubular structure from the cross-sectional image, and we estimate the neuronal fiber radius. Specifically, the skeleton point *p*_3_ and its parent point *p*_*f*_ and child point *p*_*c*_ can be approximately considered to be in the same circle with a very large radius. Moreover, the normal vector ${\overset{\rightharpoonup }{\upsilon}}_3$ of this circle at *p*_3_ can be regarded as parallel to $\overrightarrow{p_f{p}_{rc}}$ (the topological graph is shown in the supplementary materials, Figure S1 C). Subsequently, the cross-sectional plane at *p*_3_ is defined by the normal vector ${\overset{\rightharpoonup }{\upsilon}}_3$, named *Area*_3_. With regard to *Area*_3_, we segment the neuronal fiber tubular structure with a series of image processing operations, including linear grayscale transformations, adaptive thresholding [28], open operations, and connected component operations on the cross-sectional image. Finally, we obtain the inner part of the cross-section of the neuronal fiber structure at *p*_3_, named *Area*_*t*_. Here, we estimate this segmented result with a circle and estimate the radius according to the formula of a circle $r=\sqrt{S/\pi }$, where *S* = *Area*_*t*_.

### Statistical analysis

The statistical significance analysis was performed using MATLAB, and statistical comparisons were performed using a two-tailed t-test. All measurements are listed in the form *mean* ± *std*. The confidence level was set to 0.05 (*p < 0.05, **p < 0.01, ***p < 0.0001).

## Results

### The performance of the method on synthetic neuron fiber images

To verify that our method works as intended, we apply our method to synthetic images in different situations (Fig. 2). We verified three cases, including an ideal neuron fiber without any interference, noisy images, and a case where other neuron fibers are near the target neuron.

According to the results of the first case (Fig. 2b), the SSD of the optimized neuron skeleton is smaller than that of the original neuron skeleton. After making adjustments with the fiber property, the minimum reciprocal distance is not larger than 1 voxel. When the minimum reciprocal distance is set to 0 and 0.5 voxels, the SSD of the skeleton adjusted by the fiber property is slightly smaller than the SSD of the neuron skeleton adjusted by the grayscale value. This means that after optimization, the difference between the traced skeleton and the ground truth is reduced.

In whole-brain three-dimensional images, there is considerable noise. To simulate this case, we test our method under different signal-to-noise ratios (SNRs, Fig. 2c and d). The results show that the SSD increases with decreasing SNR. When the SNR is larger than 5.2 dB, the SSD of the optimized neuron skeleton is smaller than that of the original skeleton, and this trend changes slowly. However, when the SNR is lower than 5.2 dB, the SSD of the optimized neuron skeleton suddenly increases. These results suggest that our method can work well with SNRs larger than 5.2 dB. The images from the reported whole-brain imaging system [9, 10] are sufficient for our method to work.

In neuron tracing research, distinguishing two neuron fibers that are close to each other is a very difficult situation, and we test our method in such simulations. The results (Fig. 2e and f) show that the SSD of the optimized skeleton first increases and then decreases, and the SSD reaches a peak at a distance of 6 voxels between two neuron fibers. From the cross-sections of the neuron fibers in Fig. 2e, if the distance between the two neuron fibers is less than 6 voxels, it is difficult to visually distinguish the boundary of the two neuron fibers. In this case, our method performs worse than semimanual tracing. If the distance between two neuron fibers is greater than 6 voxels, the boundary can be roughly distinguished by humans. In this case, the SSD of the optimized skeleton is similar to that of the semimanually traced skeleton. This result suggests that our method does not perform very well in distinguishing adjacent neurons. If humans cannot distinguish the boundaries of nearby neuron fibers, our method will be severely affected by adjacent neuron fibers. If humans can distinguish the boundaries of adjacent neuron fibers, the accuracy of our methods is not much different from the accuracy of semimanual tracing. Although our method is not better than semimanual tracing when dealing with adjacent neuron fibers, the skeleton processed by the machine is more objective and repeatable.

In real applications, adjacent neuronal fibers often have different signal intensities compared with the target neuronal fiber. We set different grayscales for adjacent neuronal fibers in the synthetic data and tested our method on these data as shown in Figure S2 in the additional file. The results show that when the difference in signal strength between two adjacent neuronal fibers is great, the SSD score of our method is low, which indicates that our method performs well. In Fig. 2f, the two neuronal fibers have the same signal intensity, which is an extreme situation for our method.

### Applicability to the fibers of pyramidal neurons

For pyramidal neurons with long-projection axonal fibers, researchers prefer semimanual tracing. Due to the great fiber length of this type of neuron, slight inaccuracies accumulate and increase, which may cause large deviations.

**Fig. 3:**
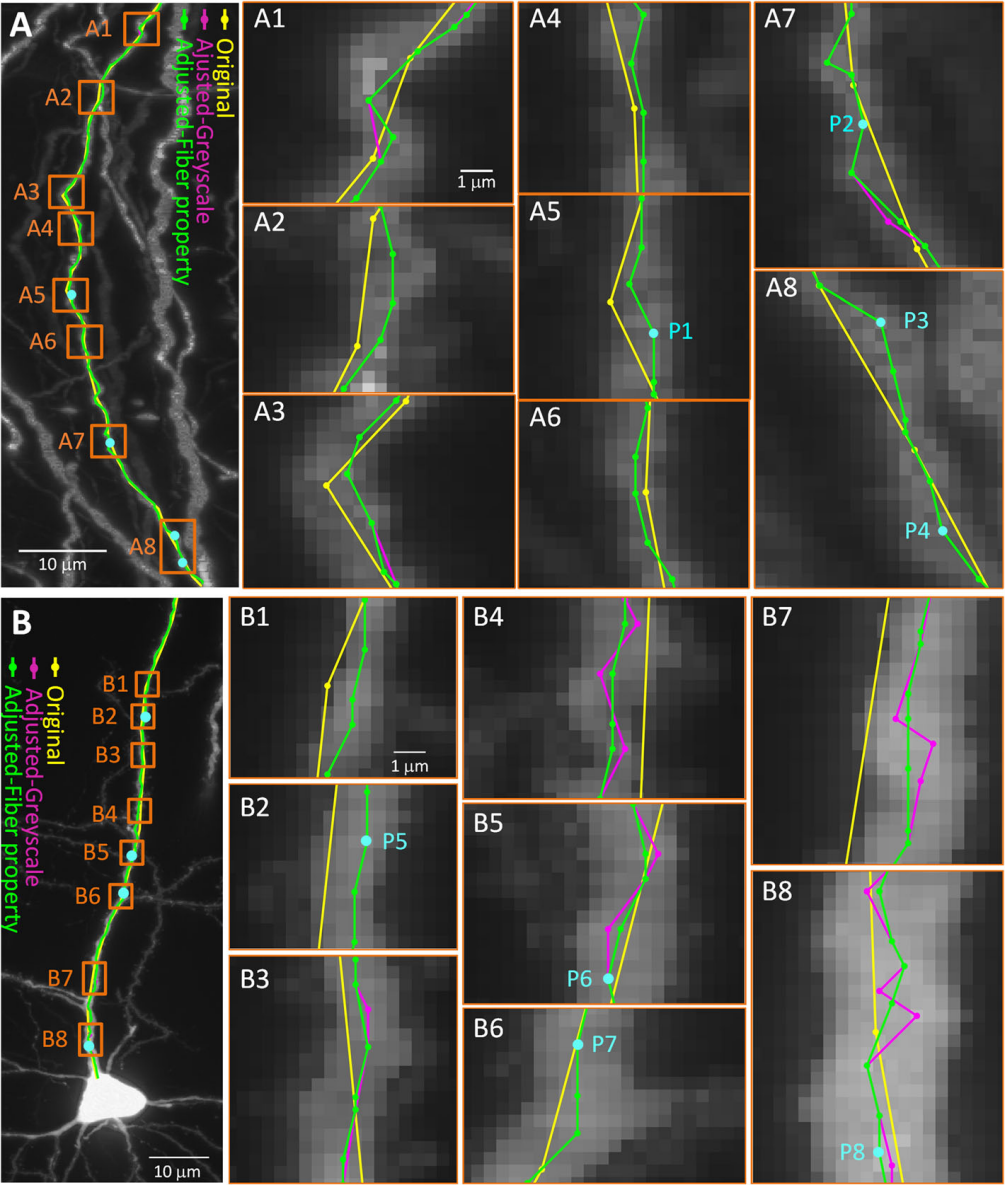
Applicability of skeleton optimization to fibers of pyramidal neurons. Skeleton of axonal (A) and dendritic (B) fibers. A1-A8 are enlarged views marked with orange blocks in A. Yellow indicates the original skeleton. Magenta indicates the skeleton after the adjustment based on grayscale, similar to B1-B8. Green indicates the skeleton after adjustment based on the neuronal shape restrictions. A2-A8 have the same scale bar as A1, and B2-B8 have the same scale bar as B1. The light blue dots (P1-P8) are the nodes whose cross-sections of the neuron fiber are shown in Fig. 5 and in the supplementary Figure S2

In applying this method to dendritic and axonal fibers, the image dataset comes from the fMOST imaging system. Figure 3 shows the axonal and dendritic fiber adjustment results for a pyramidal neuron in the mouse motor cortex. From Fig. 3A and B, the original skeletons describe the general direction of the neuron fibers (Fig. 3, yellow line). The enlarged images (Fig. 3A1-A8 and B1-B8) show that it is necessary to improve the accuracy of the traced skeletons to describe the tortuous shape of these neuron fibers. After the linear interpolation and adjustment based on grayscale, the number of skeleton points increases, and the tortuous morphology becomes more accurate (Fig. 3, magenta line). However, after grayscale adjustment, a jagged line appears in the skeleton (magenta line, Fig. 3, B4, B7 and B8). After the adjustment, based on the shape restrictions, the skeletons become smoother and match the centerline of the neuron fibers in the images more closely (Fig. 3, green line).

For long-projection neuron fibers in whole-brain images, Fig. 5A shows the results. In Fig. 5A2-A4, the optimized skeleton matches the centerline of the fibers from the images more closely.

### Applicability to the fibers of chandelier cells

**Fig. 4:**
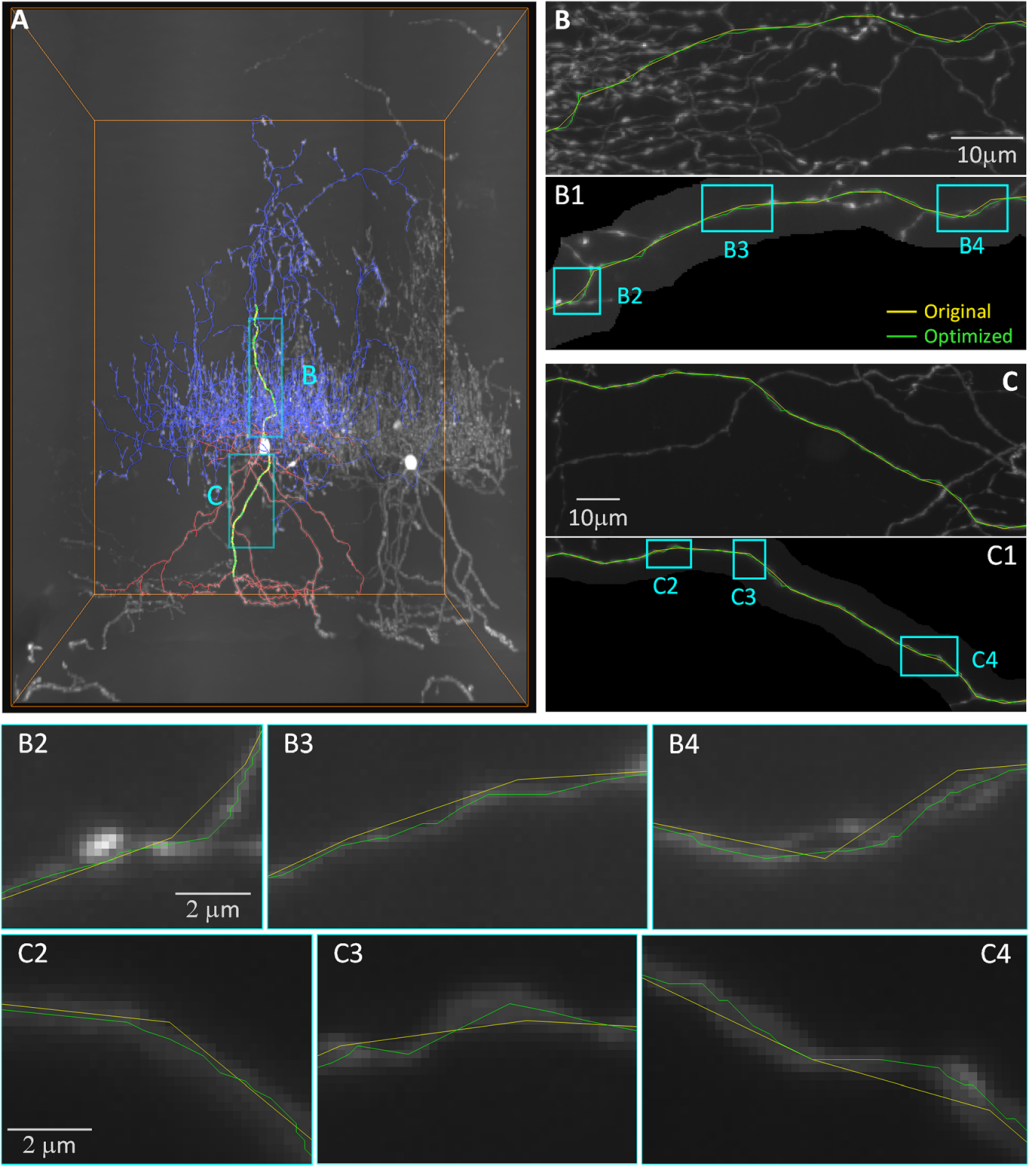
Applicability of skeleton optimization to the fibers of chandelier cells. (A) A traced chandelier cell skeleton matched with three-dimensional images. The axonal fiber is color-coded in blue, and the dendritic fiber is color-coded in red. Skeleton of chandelier axonal (B) and dendritic (C) fibers. To show the fiber clearly, the voxels around the reconstructed skeletons are abstracted in B1 and C1. B2-B4 are enlarged views marked with orange blocks in B1, similar to C2-C4. B2-B4 have the same scale bar, similar to C2-C4

Chandelier cells are GABAergic interneurons whose axonal fibers are slender and tortuous. Because their axon arbors are extremely dense and complex, researchers prefer semimanual tracing to ensure the correctness of their topology. We choose fiber images of these neurons as a representative case to test our method. Figure 4 shows the axonal and dendritic fiber optimized results for a chandelier cell. Compared with the pyramidal neuron fibers in Fig. 3, the axonal fibers of the chandelier cell are thinner and more tortuous. Although in Fig. 4A and B, the axonal fibers seem very dense, after we remove the voxels near the axonal fibers from the three-dimensional image (Fig. 4B1), the axonal fibers look less difficult for machines to identify.

In Fig. 4B1 and C1, the traced skeletons describe the general direction of the fibers (Fig. 4, yellow line). However, the enlarged images (Fig. 4B2-B4 and C2-C4) show that it is necessary to improve the accuracy of the digital skeleton to describe the tortuous shape of these dendritic and axonal fibers. After the optimization, the number of skeleton points increases, and the tortuous morphology becomes more accurate (Fig. 4, green line). Compared with the original traced skeleton, the optimized skeleton matches the centerline of the dendritic and axonal fibers from the images more closely (Fig. 4, B2-B4 and C2-C4).

### Influence of the optimized skeleton on the neuron morphological parameters

Changes in the digital neuron skeleton will inevitably have an impact on the calculation results of morphological parameters. Here, we select the neuron fiber radius, which is always calculated based on the digital neuron skeleton, and the neuron fiber path length to evaluate the performance of our method.

**Fig. 5:**
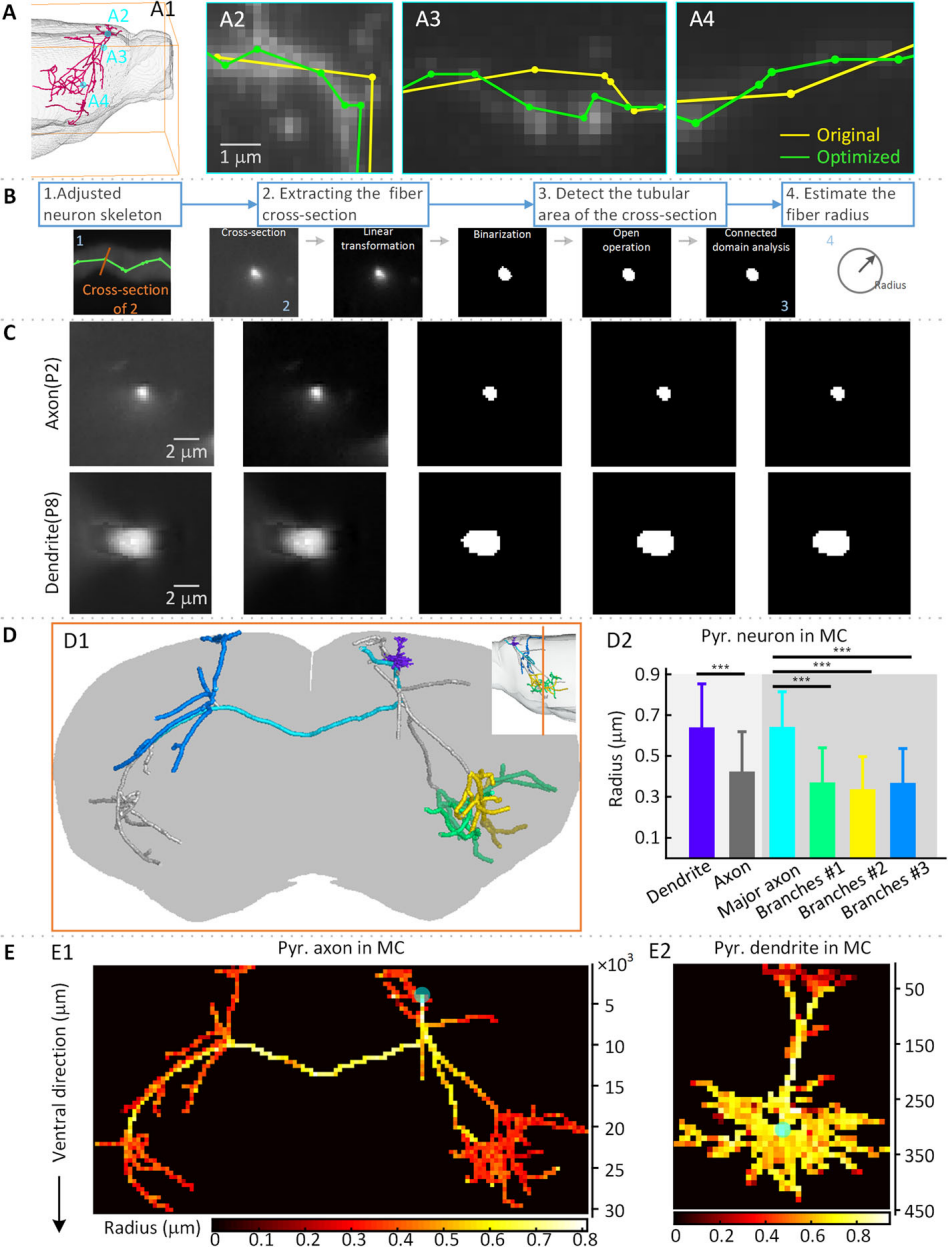
Pyramidal neuron radius distribution around the whole brain. (A) A traced neuron skeleton of a pyramidal neuron in the mouse motor cortex is optimized based on whole-brain three-dimensional images. (B) The workflow of neuron fiber estimation based on an optimized skeleton. (C) Cross-sections of a dendritic fiber and the axonal fiber reconstructed in (A). (D) The average fiber radius (D2) of different parts (color-coded in D1) of the pyramidal neuron. (E) Fiber radius distribution heat map of the axonal fiber (E1) and dendritic fiber (E2). Light blue dots represent the soma location in E

Figure 5C-E show the information of the pyramidal neuron fiber radius. According to the geometric relationship of the continuous nodes in the neuron skeleton, we extract the cross sections of the dendritic and axonal fibers (Fig. 5C; additional cross-sections are shown in the supplementary materials, Figure S3). These cross-sections show that the dendritic fiber radius is larger than the axonal fiber radius. The shape of the neuron fiber cross-sections is not a circle but an irregular ellipse. We select several fibers from different parts of the neuron, color-coded in Fig. 5D, and calculate their average fiber radius. The resulting statistic in Fig. 5D2 shows that the dendritic radius is significantly larger than the axonal radius. The radius of the major axonal fibers is significantly different from the radius of the terminal axonal branch fibers. Furthermore, we show the neuron fiber radius distribution with a heat map in Fig. 5E. These heat maps (Fig. 5E1 and E2) show that the radius of the major axonal fiber seems larger than those of the fibers branching from the major axonal fiber, and the size of the dendritic fiber radius does not show an obvious relationship with the branch order. These results are consistent with previous studies [29, 30]

The path lengths of the neuron fibers are listed in Table 1. Overall, the optimized skeletons are longer than the original skeletons. This may be caused by the optimized skeleton depicting the tortuous shape of neuron fiber in the images more accurately. In the simulation experiment, the original traced skeleton is 5.67% shorter than the ground truth. The optimized skeleton is 3.80% longer than the ground truth. This suggests that the path length of the optimized skeleton is closer to the ground truth.

**Table 1:** Branch length comparison between the traced skeleton branches and optimized skeleton branches

Fragment		Ground truth (μm)	Original (μm)	Optimized (μm)	Difference (μm)	Difference /Original
**Helix curve**		197.62	186.41	204.71	18.30	9.81%
**VIS**	Axon #1	none	3970.93	4110.62	139.69	3.52%
	Axon #2		1244.10	1264.30	20.20	1.62%
	Axon #3		952.25	980.22	27.97	2.94%
	Dendrite #1		372.70	390.53	17.83	4.78%
	Dendrite #2		115.55	119.05	3.50	3.03%
**MC**	Axon #1	none	5320.15	5536.82	216.67	4.07%
	Axon #2		1965.80	2044.52	78.72	4.00%
	Axon #3		2372.94	2404.79	31.85	1.34%
	Axon #4		3449.51	3623.17	173.66	5.03%
	Dendrite #1		161.07	176.61	15.54	9.65%
	Dendrite #2		156.35	157.24	0.89	0.57%

## Discussion

The goal of our method is to reduce the difference between the traced neuron skeleton and the centerline of the neuron fiber in images rather than correctly reconstructing the topology of the neuron skeleton. In most studies, the topology of the neuron fiber skeleton carefully traced by researchers is delicate enough to enable the projection patterns to be analyzed. Therefore, the optimized skeleton improves the accuracy of the morphological parameters. In this study, the original skeletons are semimanually traced because inaccuracy generally appears in the semimanually traced results of the long-projection neuron morphology. However, if automatically traced neuron skeletons have the same problem, it is possible to use our method to optimize the traced neuron skeleton or integrate our method into the process of neuron reconstruction to obtain a more accurate neuron fiber skeleton.

In fact, neuron fibers are not as ideal as we have seen in synthetic data. From the images of real neuron fibers in Fig. 5, the cross-sections of fibers are not circular (Fig. 5C), and the spine and bouton grow on the fibers (Fig. 5A2 and A3). These factors influence the recognition of the neuron fiber centerlines. For example, if the spine is considered to be part of a neuron fiber, the centerline may be biased toward the side where the spine exists. If not, we may have to use algorithms [31, 32] to identify these tiny structures first to avoid their influence. In this article, we optimize the skeleton without distinguishing these tiny structures. However, in the future, distinguishing these tiny structures may further improve the accuracy of the skeleton.

In our method, we use the mean shift to find the nodes on the centerline. Without the limitation of the search range, the results of the mean shift will be easily affected by noise and nearby neuron fibers. To avoid these effects, we set the search range according to the full width at half maximum of the gray distribution of a truncated line in the maximum-projection images of the neuron fibers. Therefore, when our method is used for different neuron fibers, the range must be set according to the specific situation. For example, the ranges of the axonal fibers of chandelier cells are smaller than the dendritic fibers of pyramidal neurons. On the other hand, the radius of neuron fibers is very important information for our method, especially in areas with dense fibers. After skeleton optimization, we compare the optimized skeleton with the original skeleton. If the optimized skeleton is very strange, we estimate the radius of the neuron fibers based on the original skeleton. According to the radius information, the program can optimize the traced skeleton again.

In the part of skeleton adjustment that uses shape restrictions, the reason we choose the property of neuron fibers to fine-tune the neuron skeleton is that neuron fiber shapes are variable, but this property is stable locally. In the fine-tuning step, there are two main thresholds: the angle threshold and grayscale threshold. The values of these two thresholds are adjustable. In the method section, the angle threshold is set to 135°. However, for tortuous neuron fibers such as the axonal fibers of chandelier cells, this threshold has to be set to a smaller value. For the grayscale threshold, if the local signal intensity varies greatly, the ranges of the gray degree (formula 2) have to be set to larger values. We use the angle threshold and grayscale threshold to limit the adjusting range of the skeleton nodes in a more detailed way. According to different images of the neuron fibers, these thresholds may have to be adjusted. In the methods section, we give the reference values of these thresholds, which does not mean that the values are suitable for all images.

In the radius estimation, we use only a cross-section to calculate the fiber radius. Although we use the adaptive method [28] to set the threshold for image binarization in different cross-sections, the impacts of noise and signal intensity variation cannot be avoided completely. In addition, in previous studies, images of the same neuron type labeled with different methods showed different neuron fiber radii [30]. Even if the imaging system and labeling method are the same, the tiny structure will affect the radius estimation results. For example, if spines are counted as part of the dendritic fibers, the estimated radius will be larger than the radius not including the spines. In the future, it may be more appropriate to use a three-dimensional image instead of a cross-section to estimate the radius, and more information may contribute to the robustness to noise or the signal intensity variation. In contrast, in this study, we used a circle to approximate the neuronal fiber tubular structure from cross-sectional images. However, from the cross-sectional images shown in the results, the shape of the tubular fiber is more like an irregular ellipse shape. In the future, a more complex model must be considered in order to describe neuronal fiber tubular structures.

On the other hand, in our experiments, the adjustment-based fiber property step helps to reduce the jagged lines introduced by adjustments based on grayscale. In our method, we take voxels as the minimum calculation unit; as a result, the optimized coordinates are integers. These integers may also introduce jagged lines. The jagged lines can be smoothed by performing Gaussian smoothing on the coordinates of the ordered skeleton nodes. When the nodes are evenly distributed around the ground truth, the performance of Gaussian smoothing is very good. However, when the nodes are distributed on one side of the line, Gaussian smoothing will pull the line away from the ground truth. At present, we have not completely clarified the situations when Gaussian smoothing is not effective. To ensure the stability of the algorithm, we have not yet added Gaussian smoothing in skeleton optimization. In the future, we believe that after clarifying these uncertain situations, adding Gaussian smoothing to neuron skeleton optimization will further improve the accuracy of the skeletons.

## Conclusion

In conclusion, this method focuses on improving the accuracy of the traced skeletons of neuronal fibers after neuron tracing instead of improving the completeness and correctness of neuron tracing. In the skeleton optimization stage, we take advantage of the neuronal fiber property shown in images and design shape restrictions and an evaluation index for the adjustment computation. With this method, the accuracy of the digital skeleton is improved. Compared with the morphology obtained without optimization, the optimized neuronal fiber is longer. Furthermore, based on the more accurate neuronal morphology, we analyze the neuronal fiber radius distribution and find that the major branches are thicker than the fibers split from major branches. In the future, we believe that with this method, more accurate digital skeleton data will improve the results of neuron modeling.

## Supplementary information

**Additional file 1: Figure S1.** The topological graph of skeleton points. **Figure S2.** Quantitative performance of skeleton optimization method on synthetic datasets with different signal intensity. **Figure S3.** The cross sections of axonal and dendritic fibers of pyramidal neuron.

## Data Availability

All the image datasets supporting the conclusions of the article are available, and can be obtained via contacting the corresponding author A. Li (aali@mail.hust.edu.cn) or found at http://atlas.brainsmatics.org/a/jiang2004. The codes of the method are also available via above webpage.

## Abbreviations

VIS: Visual cortex
MC: Motor cortex
fMOST: Fluorescence micro-optical sectioning tomography
SNR: Signal to noise ratio
SSD: Significant spatial distance
MD: Minimal distance
PSF: Point spread function
