# Supplementary material for: Skeleton optimization of neuronal morphology based on three-dimensional shape restrictions
Source: BMC Bioinformatics. 2020 Sep 4;21:395. doi: 10.1186/s12859-020-03714-z (PMC7472589; doi:10.1186/s12859-020-03714-z)
Supplement: Supplementary file 1 — Additional file 1: Figure S1. The topological graph of skeleton points. Figure S2. Quantitative performance of skeleton optimization method on synthetic datasets with different signal intensity. Figure S3. The cross sections of axonal and dendritic fibers of pyramidal neuron. [file 12859_2020_3714_MOESM1_ESM.pdf]

Supplementary Materials

Skeleton optimization of neuronal morphology based on three-dimensional shape restrictions

Siqi Jiang[1], Zhengyu Pan[1], Zhao Feng[1], Yue Guan[1], Miao Ren[3], Zhangheng Ding[1], Shangbin Chen[1], Hui Gong[1, 2, 4], Qingming Luo[1, 2, 3], Anan Li[1, 2, 4]*

[1] *Britton Chance Center for Biomedical Photonics, Wuhan National Laboratory for Optoelectronics, MoE Key Laboratory for Biomedical Photonics, School of Engineering Sciences, Huazhong University of Science and Technology, Wuhan, China*

[2] *HUST-Suzhou Institute for Brainsmatics, JITRI Institute for Brainsmatics, Suzhou, China*

[3] *School of Biomedical Engineering, Hainan University, Haikou, China*

[4] *CAS Center for Excellence in Brain Science and Intelligence Technology, Chinese Academy of Science, Shanghai, China*

*Corresponding author. Email: aali@mail.hust.edu.cn (Li A)

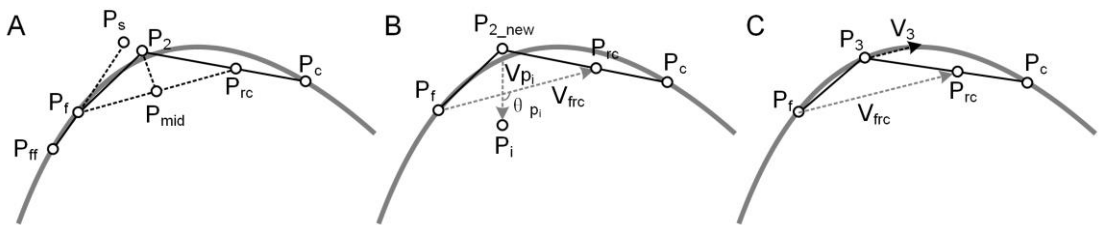

Figure S1 The topological graph of skeleton points. (A) The topological relationships of points related to the point P_{2_new}. (B) The topological relationships of points related to the new skeleton point, named P_3. (C) The topological relationships of the skeleton points related to the neuronal fiber radius estimation.

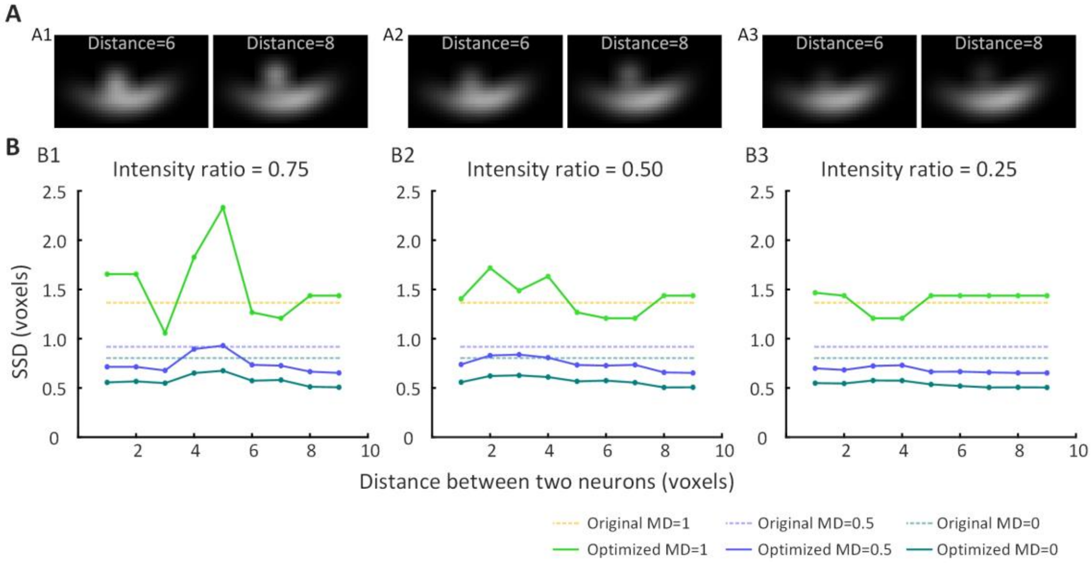

Figure S2 Quantitative performance of skeleton optimization method on synthetic datasets with different signal intensity. The grayscale of voxels corresponding to the helix curve is set to 255, and the grayscale of the adjacent neuronal voxels is set to 0.75, 0.5 and 0.25 times of 255. (A) The cross sections of synthetic neuron fiber with different signal intensity. A1-A3 correspond to signal intensity of 0.75, 0.5 and 0.25 times of 255 respectively. (B) Skeleton optimization performance on dataset with different signal intensity. B1-B3 correspond to the SSD using signal intensity of 0.75, 0.5 and 0.25 times of 255 respectively. Comparing the SSD in B1, B2 and B3, when the intensity ratio is smaller, the adjacent neuronal fiber is easier to identify, and the SSD score is smaller, which means the method performance is better. Judging from the SSD when minimum distance is set to 1 voxel, the method performance is consistent with the situation shown in the Figure 2 F.

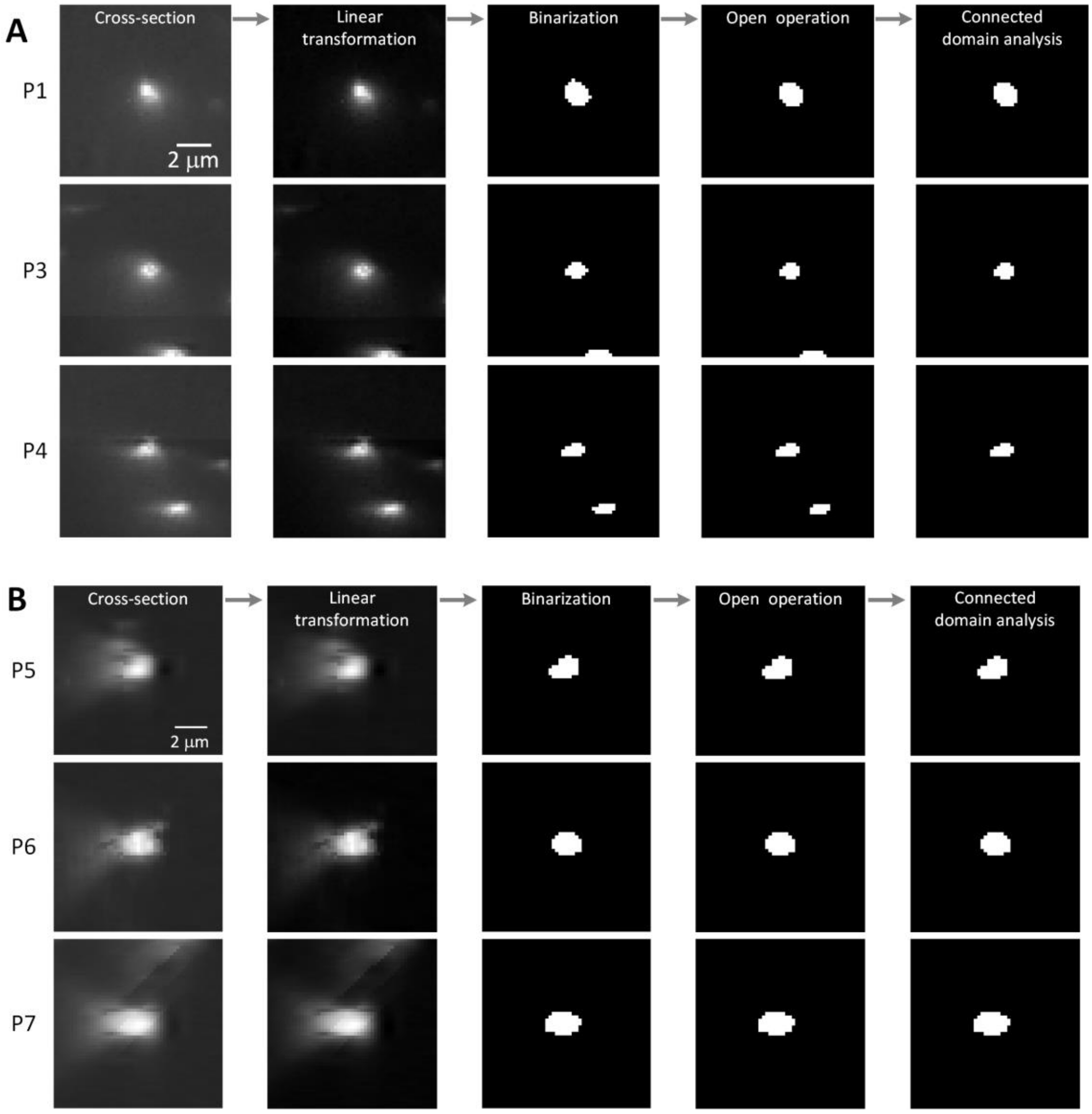

Figure S3 The cross sections of axonal (A) and dendritic (B) fibers of pyramidal neuron.
